# The genetic structure of *Arabidopsis thaliana* in the south-western Mediterranean range reveals a shared history between North Africa and southern Europe

**DOI:** 10.1186/1471-2229-14-17

**Published:** 2014-01-10

**Authors:** Adrian C Brennan, Belén Méndez-Vigo, Abdelmajid Haddioui, José M Martínez-Zapater, F Xavier Picó, Carlos Alonso-Blanco

**Affiliations:** 1 Estación Biológica de Doñana (EBD), Consejo Superior de Investigaciones Científicas (CSIC), Seville, Spain; 2 Centro Nacional de Biotecnología (CNB), Consejo Superior de Investigaciones Científicas (CSIC), Madrid, Spain; 3 Faculté des Sciences et Techniques, Université Sultan Moulay Slimane, Beni Mellal, Morocco; 4 Instituto de Ciencias de la Vid y del Vino (Consejo Superior de Investigaciones Científicas, Universidad de La Rioja, Gobierno de La Rioja), Logroño, Spain

**Keywords:** *Arabidopsis thaliana*, Population genetics, Natural variation, Genetic diversity, Genetic structure, Demographic history, North Africa, Mediterranean Basin, Glacial refugium/refugia

## Abstract

**Background:**

Deciphering the genetic structure of *Arabidopsis thaliana* diversity across its geographic range provides the bases for elucidating the demographic history of this model plant. Despite the unique *A. thaliana* genomic resources currently available, its history in North Africa, the extreme southern limit in the biodiversity hotspot of the Mediterranean Basin, remains virtually unknown.

**Results:**

To approach *A. thaliana* evolutionary history in North Africa, we have analysed the genetic diversity and structure of 151 individuals collected from 20 populations distributed across Morocco. Genotyping of 249 genome-wide SNPs indicated that Morocco contains substantially lower diversity than most analyzed world regions. However, IBD, STRUCTURE and PCA clustering analyses showed that genetic variation is strongly geographically structured. We also determined the genetic relationships between Morocco and the closest European region, the Iberian Peninsula, by analyses of 201 populations from both regions genotyped with the same SNPs. These analyses detected four genetic groups, but all Moroccan accessions belonged to a common Iberian/Moroccan cluster that appeared highly differentiated from the remaining groups. Thus, we identified a genetic lineage with an isolated demographic history in the south-western Mediterranean region. The existence of this lineage was further supported by the study of several flowering genes and traits, which also found Moroccan accessions similar to the same Iberian group. Nevertheless, genetic diversity for neutral SNPs and flowering genes was higher in Moroccan than in Iberian populations of this lineage. Furthermore, we analyzed the genetic relationships between Morocco and other world regions by joint analyses of a worldwide collection of 337 accessions, which detected an additional weak relationship between North Africa and Asia.

**Conclusions:**

The patterns of genetic diversity and structure of *A. thaliana* in Morocco show that North Africa is part of the species native range and support the occurrence of a glacial refugium in the Atlas Mountains. In addition, the identification of a genetic lineage specific of Morocco and the Iberian Peninsula indicates that the Strait of Gibraltar has been an *A. thaliana* migration route between Europe and Africa. Finally, the genetic relationship between Morocco and Asia suggests another migration route connecting north-western Africa and Asia.

## Background

*Arabidopsis thaliana* is a wild, annual, self-fertilizing plant with a broad geographic range as a native species in the Eurasian continent [1,2]. In the past decade, this species has become the main model plant, not only for molecular biology studies [3] but also for addressing the ecological and evolutionary bases of plant adaptation [4-6]. Deciphering the genetic structure of *A. thaliana* diversity across its geographic range is now a major aim because it explains its current ecological distribution, it reflects its demographic history, and it enables the precise design and analysis of experimental populations used to determine the molecular mechanisms of adaptive traits [6-8].

Currently, more than 6000 wild genotypes (accessions) of *A. thaliana* from different world regions have been collected. Studies of the genetic diversity and structure, at global (worldwide) and regional scales, have proposed several major events in *A. thaliana* demographic history in Europe [9-14]. In particular, high diversity has been described in the Mediterranean Peninsulas compared to Central and northern Europe, hence supporting the existence of multiple Pleistocene glacial refugia in southern Europe [15,16]. Further haplotype network analyses have led to the tentative suggestion of the Caucasus region as the ancestral *A. thaliana* centre of origin [15]. However, the largest diversity has been found in the Iberian Peninsula, whose strong geographic structure has prompted the hypothesis of multiple Iberian glacial refugia with differential contribution to the colonization of Europe [16]. Various global genetic analyses have identified two main postglacial colonization routes of Europe, from at least two refugia [9,12,16,17]. A glacial refugium in the Iberian Peninsula has been proposed to contribute to a west–east colonization of western and northern Europe, while an Asian refugium was likely the source for an east–west colonization of eastern and northern Europe. In agreement with this view, genetic structure analyses of northern European populations have detected several differentiated clusters, which support multiple sources of postglacial colonization [18-20]. In addition, a few studies have addressed *A. thaliana* history in East Asia and in regions outside the Eurasian continent. Nuclear and chloroplast analyses of *A. thaliana* populations from China have also shown substantial genetic variation and geographic structure, hence suggesting a rapid *A. thaliana* west–east expansion from Central Asia [21,22]. Moreover, genetic studies of North American and Japanese populations have detected no or weak geographic patterns, which indicate a very recent colonization from multiple sources [13,23,24].

Despite the unique resources available for *A. thaliana*, the scarcity of accessions from many regions located outside of Europe has hampered our knowledge of its demographic history in Africa and Asia. For instance, the centre of origin of *A. thaliana* is still unknown, since conclusive evidence for one of the two classical hypothetical locations, Europe-North Africa and Middle Asia, requires further samples and studies from those regions [2,15]. In particular, North Africa (from Morocco to Libya) is the extreme southern limit of *A. thaliana* distribution in the Mediterranean Basin, and it is assumed to be part of the native range [1,2]. However, the history of *A. thaliana* in this region remains virtually ignored since less than a handful of African accessions have been studied until now [10,14,15].

The relevance of northern Africa in structuring biodiversity, at global and regional scales, has been well documented for several plant species (reviewed in [25]). This is determined mainly by the unique biogeographic location of this region, which is part of one of the world’s largest biodiversity hotspot, the Mediterrranean Basin, and it contains migration routes from Europe, Africa and Asia [25,26]. A small number of phylogeographical and palaeoecological studies have identified several Pleistocene glacial refugia for perennial and annual plants in the African side of the Mediterranean Basin [25]. In addition, the central-marginal hypothesis for the geographic distribution of diversity across species’ ranges predicts lower genetic diversity and higher genetic differentiation in peripheral than central populations (reviewed in [27]). However, most studies supporting these expectations, including those of *A. thaliana*, have focused in the northern limit of species from the North hemisphere [27-29]. Therefore, additional studies estimating the genetic diversity and structure of plant species, especially herb plants, in southern limits like North Africa are needed to evaluate current hypotheses explaining plant evolutionary histories and their actual geographic distributions.

In this study we have addressed the genetic diversity and structure of *A. thaliana* in North Africa by developing a new collection of accessions derived from populations distributed throughout the species range in Morocco. In particular, we aim to determine, first, if North Africa has been recently colonized, or if it might contain genetically isolated and structured populations supporting the occurrence of *A. thaliana* as a native species. Second, we aim to detect genetic relationships between Morocco and other geographic regions, which might provide information about the demographic history in North Africa. To approach these questions, we have analysed this Moroccan collection together with a collection from the nearest European region, the Iberian Peninsula, and a worldwide collection. Population genetic analyses of genome-wide neutral markers and potentially adaptive flowering genes and traits, demonstrate a shared history in southern Europe and northern Africa. Together, these analyses identified the Strait of Gibraltar as a migration route between Europe and Africa, and suggest that the Atlas area was a Pleistocene glacial refugium for *A. thaliana*.

## Results

### Genetic diversity and structure in North Africa

**Figure 1:**
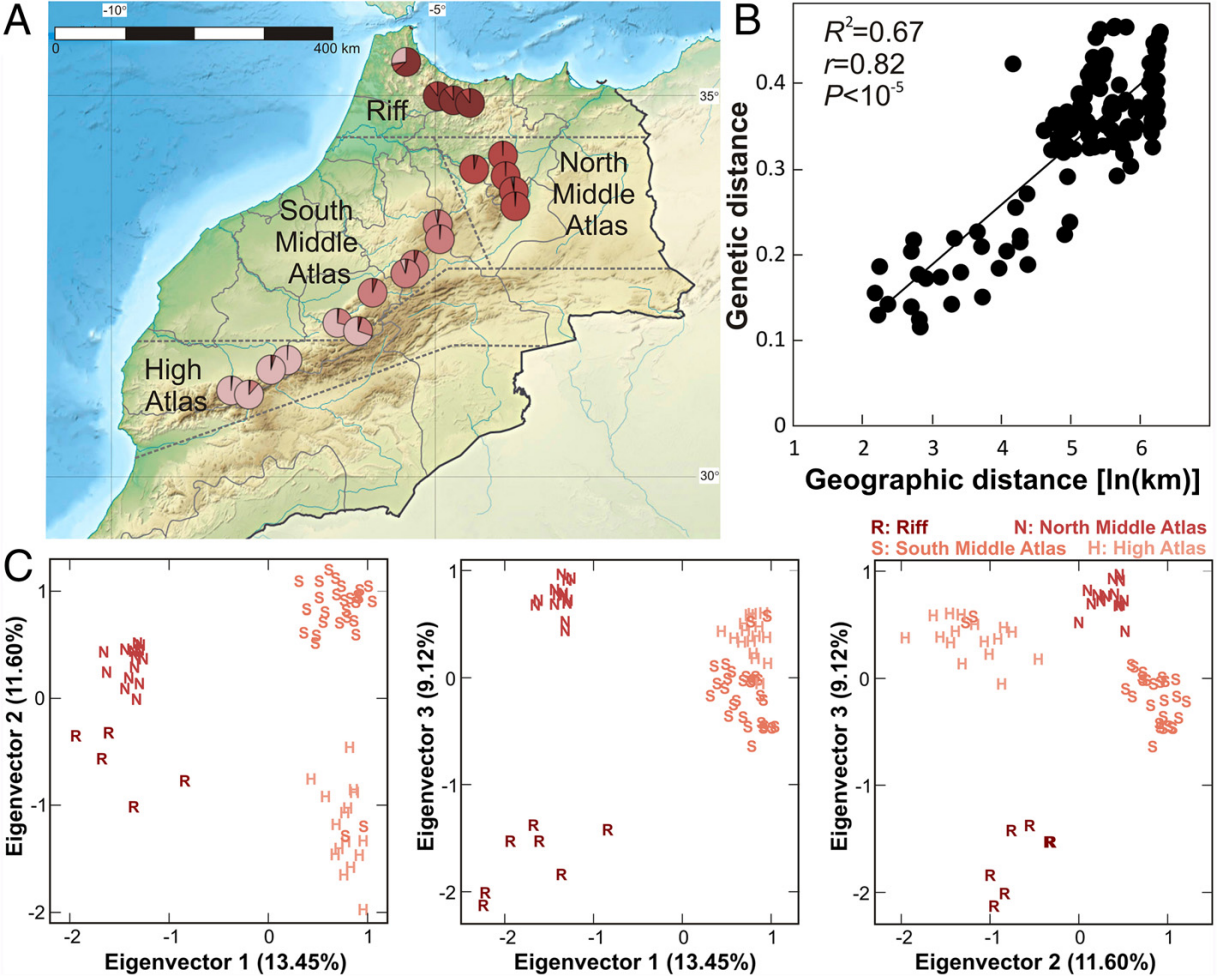
**Genetic and geographic structure of *A. thaliana *in Morocco. A)** Geographic location of Moroccan populations and their genetic relationships inferred with STRUCTURE. Each population is depicted as a pie chart quantifying membership proportions for four genetic clusters (*K* = 4). **B)** Correlation between geographic and genetic distances (average proportion of allelic differences) among populations. **C)** Scatter plots displaying paired combinations of the first three eigenvectors estimated by PCA of Moroccan genotypes. The four main groups detected by clustering analysis of these principal components are shown with the same colors as the STRUCTURE clusters of A.

To determine the genetic diversity of *A. thaliana* in North Africa we sampled 151 individuals in 20 populations distributed across the major mountain ranges of Morocco (Figure 1A, Additional file 1: Figure S1, Additional file 2: Table S1). Genotyping of this collection with a genome-wide set of 249 presumably neutral single nucleotide polymorphisms (SNPs) detected a total of 65 different haplotypes. No haplotype was shared by several populations. Moreover, substantial variation was found among populations for the number of haplotypes per population, because two populations carried a single haplotype while two others contained seven or more genotypes (Table 1). Similar variation was detected for gene diversity (*H*_*S*_) and allelic richness (*R*_*S*_) per population, which showed up to a tenfold range of variation among populations. The amount of genetic variation per population displayed a weak geographic pattern since the percentage of polymorphic loci (*PL*) correlated negatively with latitude (*r* = −0.46; *P* = 0.046), and *R*_*S*_ showed similar negative but marginal correlation (*r* = −0.4; *P* = 0.09). In agreement, four geographic subregions established according to latitude differed slightly for *PL, R*_*S*_ and *H*_*S*_ (0.08 > *P* > 0.04), populations from the Riff containing less genetic variation than populations from the High Atlas (Table 1).

**Table 1:** **Genetic diversity of *A. thaliana *** **populations from Morocco**

**Population**	**Subregion**^**1**^	***N***	***N***_***H***_	***PL***	***H***_***S***_	***R***_***S***_	***R***_***P***_
Zin	1	9	2	14	0.031±0.007	1.03±0.01	0.026±0.013
Bab	1	4	2	3	0.015±0.008	1.02±0.01	0.008±0.008
Bbe	1	10	1	0	0±0	1±0	0.003±0.003
Ket	1	8	2	5	0.018±0.007	1.02±0.01	0.008±0.008
**Mean**	1	7.8	1.8	5	0.016±0.006	1.02±0.01	0.011±0.008
Taz	2	6	4	12	0.052±0.013	1.06±0.01	0.002±0.002
Tah	2	10	2	16	0.078±0.016	1.08±0.02	0±0
Bba	2	3	3	14	0.062±0.014	1.07±0.02	0.005±0.005
Meh	2	10	3	13	0.027±0.006	1.03±0.01	0±0
Tiz	2	10	2	21	0.104±0.018	1.11±0.02	0±0
**Mean**	2	7.8	2.8	15	0.065±0.011	1.07±0.02	0.001±0.001
Ifr	3	7	5	24	0.09±0.0150	1.1±0.02	0.007±0.006
Azr	3	10	6	30	0.109±0.016	1.11±0.02	0.007±0.004
Agl	3	8	7	23	0.084±0.014	1.09±0.02	0.002±0.002
Khe	3	10	2	18	0.057±0.011	1.06±0.01	0.001±0.001
Elk	3	10	6	13	0.039±0.009	1.04±0.01	0.009±0.008
Oua	3	1	1	-	-	-	-
Til	3	10	1	0	0±0	1±0	0±0
**Mean**	3	9.2	4.5	18	0.063±0.013	1.07±0.02	0.004±0.004
Elh	4	10	10	42	0.15±0.0170	1.16±0.02	0.012±0.006
Arb	4	2	2	18	0.092±0.017	1.12±0.02	0±0
Ait	4	6	2	17	0.048±0.009	1.05±0.01	0.001±0.001
Set	4	7	2	20	0.049±0.009	1.05±0.01	0±0
**Mean**	4	6.25	4	24	0.085±0.013	1.09±0.01	0.003±0.002

The following parameters are shown: number of individuals (*N*), number of haplotypes (*N*_*H*_), percentage of polymorphic loci (*PL*), gene diversity (*H*_*S*_), allelic richness (*R*_*S*_) and private allelic richness (*R*_*P*_) per locus. Populations are arranged according to latitude, from north to south, and classified into four subregions as in Figure 1. *H*_*S*_, *R*_*S*_ and *R*_*P*_ are mean values ± SE.

^1^: 1: Riff; 2: North Middle Atlas; 3: South Middle Atlas; 4: High Atlas.

The genetic relationship among Moroccan populations was first analysed by constructing Neighbor-joining (NJ) trees of the 65 haplotypes (Additional file 1: Figure S2A), which showed that genotypes are genetically closer within than among populations. AMOVA analyses indicated that populations were highly differentiated, with 81.6% of the genetic variation appearing distributed among populations (*F*_*ST*_ range: 0.26-1) and only 18.4% occurring within populations (*P* < 0.001). Mantel tests of pair-wise geographic distances and genetic distances, measured as *F*_*ST*_ or as percentage of allele differences among populations, detected highly significant correlations (0.59 < *r* < 0.82; *P* < 0.001; Figure 1B) accounting for up to 67% of the variance. Therefore, *A. thaliana* genetic variation showed a continuous isolation by distance (IBD) geographic pattern across Morocco.

To further determine the genetic structure of *A. thaliana* in Morocco we analysed the different haplotypes using two clustering approaches, STRUCTURE and principal component (PCA) analyses. STRUCTURE analysis detected four major clusters that closely corresponded to the four geographic subregions (Figure 1, Additional file 1: Figure S2B). Clustering analyses using the first three principal components, which explained 13.5, 11.6 and 9.1% of the genetic variation, identified precisely the same four groups (Figure 1C, Additional file 2: Table S2). These genetic groups were also supported by NJ analyses since the four main NJ clades corresponded to such clusters (Additional file 1: Figure S2A). AMOVA analyses indicated that 43.1% of the genetic variation differentiates the four groups, whereas 40.2% differentiates populations within groups. Thus, *A. thaliana* genetic variation for neutral markers appears strongly geographically structured in Morocco.

### Genetic diversity and structure in the south-western Mediterranean region

To establish the genetic relationships among *A. thaliana* populations in the south-western Mediterranean range, we analysed the 20 Moroccan populations together with a set of 181 previously sampled Iberian populations [30]. Genotyping of a single random individual from each population with the same genome-wide set of SNPs showed that all 201 samples were different genotypes. Gene diversity and allelic richness values indicated that *A. thaliana* diversity in Morocco was considerably lower than in the Iberian Peninsula (Table 2). Mantel tests of correlations between geographic and genetic distances detected significantly higher correlation coefficients and slopes of linear regressions in Morocco than in Iberia (*P* = 0.02; Additional file 2: Figure S3). However, the difference between these slopes was mainly determined by the larger genetic distances between geographically closer populations of Iberia. Therefore, the apparently stronger IBD pattern detected in Morocco might reflect higher Iberian diversity or a more complex Iberian geographic structure at lower spatial scales.

**Table 2:** **Genetic diversity of *A. thaliana *** **in different world regions**

**World region**	***N***	***PL***	***H***_***S***_	***R***_***S***_	***R***_***P***_
North America	6	66	0.266±0.013	1.66±0.03	0±0
Morocco	20	54	0.192±0.013	1.50±0.03	0±0
Iberia	181	95	0.315±0.011	1.78±0.02	0.015±0.004
British Isles	11	81	0.299±0.012	1.77±0.02	0.005±0.003
Central Europe	51	92	0.324±0.011	1.79±0.02	0.003±0.001
Fennoscandia	13	79	0.308±0.012	1.76±0.03	0.004±0.003
South Europe	7	72	0.284±0.013	1.72±0.03	0.003±0.003
East Europe	6	63	0.247±0.013	1.63±0.03	0±0
East Asia	8	73	0.279±0.012	1.72±0.03	0.001±0.000
Caucasus	10	71	0.276±0.013	1.70±0.03	0.005±0.003
Central Asia	8	79	0.298±0.011	1.78±0.03	0.007±0.005
Japan	12	73	0.277±0.012	1.70±0.03	0.002±0.001

The following parameters are shown for world regions arranged from west to east: number of populations (*N*), percentage of polymorphic loci (*PL*), gene diversity (*H*_*S*_), allelic richness (*R*_*S*_) and private allelic richness (*R*_*P*_) per locus. *H*_*S*_, *R*_*S*_ and *R*_*P*_ are mean values ± SE.

**Figure 2:**
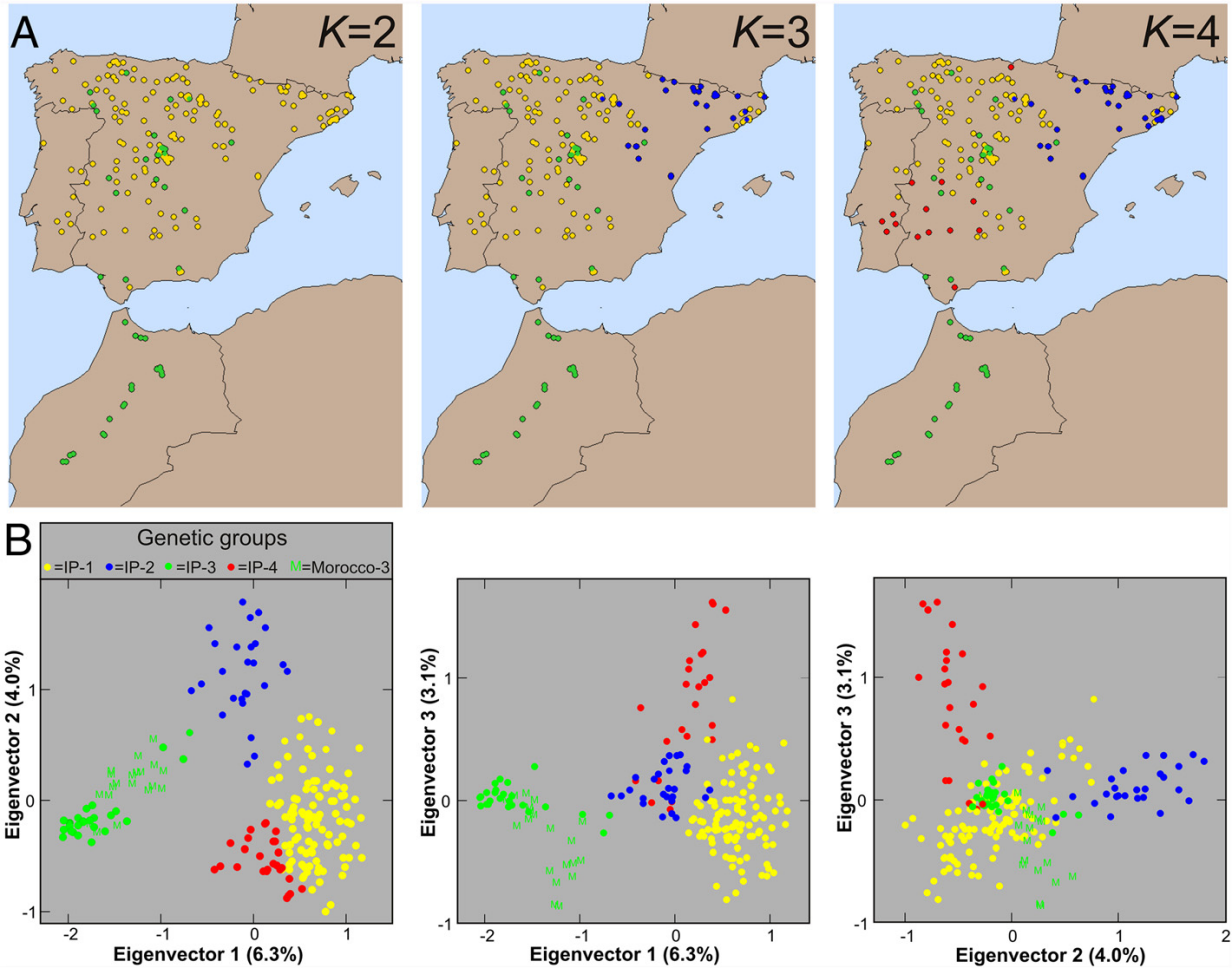
**Genetic and geographic structure of *A. thaliana *in the south-western Mediterranean region. A)** Genetic group assignment of Moroccan and Iberian accessions based on majority membership proportions inferred with STRUCTURE, for *K* = 2, *K* = 3 and *K* = 4. **B)** Scatter plots displaying pair combinations of the first three eigenvectors estimated by PCA of Moroccan and Iberian genotypes. The four main groups detected by clustering analysis of these principal components are shown with the same colors as STRUCTURE clusters of A, except that genetic group 3 is divided into Iberia (IP-3) and Morocco (Morocco-3).

The genetic structure in this region was further analysed with STRUCTURE and PCA clustering methods. STRUCTURE analyses detected four different genetic groups showing distinct geographic distribution (Figure 2A, Additional file 1: Figure S4). Clusters were numbered as described in a previous study with a lower number of populations and SNPs [16], groups 1, 2 and 4 appearing distributed mainly in NW, NE and SW of the Iberian Peninsula. By contrast, group 3 displayed a broader geographic distribution because it included 24 Iberian accessions scattered throughout this region, as well as all Moroccan genotypes. The first three principal components detected by PCA accounted for 6.5, 4.1 and 3.1% of the genetic variation. Clustering analyses of these three components identified very similar groups to STRUCTURE (Figure 2B) since 92% of the accessions were assigned to the same group based on the major cluster membership of each accession (Additional file 2: Table S2). AMOVA analyses showed an average *F*_*ST*_ differentiation among Iberian genetic groups of 0.28. However, group 3 displayed the largest differentiations (0.28 < *F*_*ST*_ < 0.45; *P* < 0.001) in agreement with the early distinction of this cluster in the first (*K* = 2) analysis of STRUCTURE (Figure 2A). In addition, low differentiation (*F*_*ST*_ = 0.24) and low mean allelic differences (12.3%) were estimated between Iberian and Moroccan groups of accessions belonging to cluster 3. Genetic diversity measurements also showed that group 3 contained the lowest amount of variation, although Morocco accessions contained higher diversity than Iberian accessions of this group (Table 3). Together, these analyses indicated that *A. thaliana* populations from Morocco are genetically related to populations assigned to group 3 from the Iberian Peninsula.

**Table 3:** **Genetic diversity in different *A. thaliana *** **clusters detected in Morocco and Iberia, and in worldwide analyses**

**Analysis**	**Genetic cluster**^**1**^	***N***	***PL***	***H***_***S***_	***R***_***S***_	***R***_***P***_
Morocco and Iberia	Morocco	20	57	0.202±0.013	1.52±0.03	0.008±0.003
	1	112	98	0.321±0.011	1.81±0.02	0.051±0.009
	2	31	85	0.273±0.012	1.72±0.02	0.041±0.009
	3	24	58	0.145±0.011	1.45±0.03	0.005±0.003
	4	14	64	0.195±0.012	1.57±0.03	0.015±0.005
Worldwide	1	159	95	0.311±0.011	1.78±0.02	0.016±0.004
	2	34	83	0.271±0.012	1.71±0.02	0.029±0.008
	3	28	63	0.147±0.010	1.46±0.03	0.003±0.002
	4	14	67	0.203±0.012	1.59±0.03	0.008±0.004
	5	68	93	0.329±0.011	1.81±0.02	0.033±0.007
	6	34	73	0.235±0.012	1.61±0.03	0.003±0.002

The following parameters are shown for the genetic groups detected by STRUCTURE analyses of Iberian and Moroccan accessions and of all worldwide accessions: number of populations (*N*), percentage of polymorphic loci (*PL*), gene diversity (*H*_*S*_), allelic richness (*R*_*S*_) and private allelic richness (*R*_*P*_) per locus. *H*_*S*_, *R*_*S*_ and *R*_*P*_ are mean values ± SE.

^1^: Genetic group 3 is divided into Moroccan and Iberian accessions in the top analysis.

### Diversity and differentiation for flowering genes, traits and climatic variables in the south-western Mediterranean region

The genetic diversity of *A. thaliana* in the south-western Mediterranean range was also analysed for the nucleotide variation of four well-known flowering genes that are likely involved in adaptation (Methods). Overall, flowering genes showed larger diversity in Iberia than Morocco as estimated by the private allelic richness (Table 4). Genes showed similar nucleotide diversity patterns in Morocco and Iberia, with *FRI* and *FLC* displaying low silent diversities and *CRY2* and *PHYC* high values (Table 4). However, analyses of the major known functional polymorphisms for these genes showed a lower frequency of predicted *FRI* loss-of-function mutations in Moroccan than Iberian accessions. In addition, as previously reported in Iberia, only one of the two major European haplogroups described in *FLC* was detected in Morocco (Table 4). Furthermore, the two major European haplogroups of *CRY2* and *PHYC* were also found in Morocco at similar low frequencies to the Iberian Peninsula (Table 4). Interestingly, a new *CRY2* haplogroup named as *CRY2*-C, differentiated by three aminoacid substitutions, was found at high frequency in Morocco (35%). This *CRY2* haplogroup appeared at very low frequency in Iberia (1.1%) and explained the larger Moroccan *CRY2* variation. Therefore, *A. thaliana* populations from Morocco showed a similar amount and pattern of diversity to the Iberian Peninsula for *FLC* and *PHYC*. However, Moroccan populations displayed higher and lower amounts of potentially functional diversity at *CRY2* and *FRI,* respectively.

**Table 4:** Genetic diversity of flowering genes in Morocco and Iberia

**Gene**	**Sequence length**^**1**^	**Genetic group**^**2**^	**Number of populations**	**Number of polymorphisms**^**3**^	***N***_***H***_^**3**^	***H***_***S***_^**3**^	***R***_***S***_^**3**^	***R***_***P***_^**3**^	***π***_***silent***_	**Haplogrups or truncations**^**4**^	**Haplogrup frequency (%)**^**4**^
*FRI*	3470	M	20	24	16	0.049±0.011	1.18±0.03	0.092±0.022	0.0023	Yes	5
		IP	178	87	97	0.058±0.009	1.24±0.03	0.181±0.023	0.0023	Yes	12.2
		IP-1	111	69	54	0.059±0.009	1.24±0.03	0.079±0.011	0.0023	Yes	17.9
		IP-2	29	26	14	0.029±0.007	1.13±0.02	0.028±0.010	0.0010	Yes	6.5
		IP-3	24	35	22	0.055±0.011	1.21±0.03	0.098±0.018	0.0026	No	0
		IP-4	14	19	7	0.046±0.011	1.15±0.03	0.032±0.014	0.0019	No	0
*FLC*	451	M	20	4	5	0.126±0.058	1.31±0.13	0.314±0.135	0.0033	A	100
		IP	181	8	18	0.104±0.035	1.38±0.11	0.378±0.106	0.0039	A	100
		IP-1	112	7	8	0.081±0.032	1.31±0.11	0.144±0.092	0.0032	A	100
		IP-2	31	3	3	0.073±0.040	1.22±0.12	0.064±0.041	0.0022	A	100
		IP-3	24	4	4	0.047±0.028	1.19±0.09	0.083±0.072	0.0025	A	100
		IP-4	14	2	3	0.049±0.039	1.14±0.10	0.106±0.078	0.0027	A	100
*CRY2*	1529	M	20	8	6	0.104±0.036	1.29±0.09	0.210±0.078	0.0229	A, B	B=5
		IP	181	19	24	0.054±0.023	1.20±0.06	0.139±0.047	0.0145	A, B	B=9.9
		IP-1	112	10	8	0.037±0.021	1.13±0.05	0.029±0.011	0.0075	A, B	B=3.6
		IP-2	31	5	5	0.033±0.022	1.10±0.05	0.031±0.021	0.0052	A, B	B=3.2
		IP-3	24	7	8	0.052±0.024	1.18±0.07	0.162±0.064	0.0023	A	100
		IP-4	14	4	3	0.052±0.025	1.16±0.08	0.051±0.044	0.0229	A, B	B=85.7
*PHYC*	868	M	19	6	7	0.065±0.033	1.24±0.11	0.121±0.059	0.0168	L*er*, Col	Col=5
		IP	160	18	34	0.062±0.024	1.23±0.06	0.141±0.036	0.0106	L*er*, Col	Col=7.4
		IP-1	102	13	15	0.061±0.024	1.23±0.06	0.090±0.032	0.0092	L*er*, Col	Col=6.9
		IP-2	30	3	4	0.026±0.018	1.09±0.05	0.032±0.029	0.0039	L*er*	100
		IP-3	16	8	9	0.070±0.028	1.25±0.08	0.100±0.045	0.0158	L*er*, Col	Col=16.7
		IP-4	12	5	6	0.062±0.028	1.20±0.11	0.035±0.024	0.0148	L*er*, Col	Col=14.3

For each gene, the following parameters are shown for Morocco, the Iberian Peninsula and the four Iberian genetic groups: number of polymorphisms, number of haplotypes (*N*_*H*_), gene diversity (*H*_*S*_), allelic richness (*R*_*S*_), private allelic richness (*R*_*P*_), silent nucleotide diversity (*π*_*silent*_), and the type and frequency of major haplogroups or functional alleles. *H*_*S*_, *R*_*S*_ and *R*_*P*_ are mean values ± SE.

^1^: Alignment length of all sequences.

^2^: Genetic groups correspond to the four main Iberian groups detected with STRUCTURE; IP: Iberian Peninsula; M: Morocco.

^3^: Only polymorphisms that are not in complete LD are included.

^4^: Accessions are classified as functional and loss-of-function truncations for *FRI*[30] or as the two major functional haplogroups described for *FLC*, *CRY2* and *PHYC*[54-56]. Frequencies are given for truncations and for the minor frequency haplogroups.

The genetic relationships between Morocco and each of the four Iberian genetic groups detected with neutral markers were also analysed using flowering gene sequences. Gene diversity and allelic richness of Moroccan populations were most similar to those of Iberian genetic group 3, although Moroccan diversities were mostly higher (Table 4). In addition, in contrast to Morocco, Iberian group 3 was not segregating for *FRI* loss-of-function alleles and for the major *CRY2* haplogroups (Table 4). Pair-wise analyses of *F*_*ST*_ values between Morocco and the four Iberian genetic groups showed that group 3 displays the lowest genetic differentiation from Moroccan populations for most genes (0.04 < *F*_*ST*_ < 0.27; Additional file 2: Table S3). Hence, *A. thaliana* populations from Morocco appeared also most similar to Iberian genetic group 3 for flowering genes.

**Figure 3:**
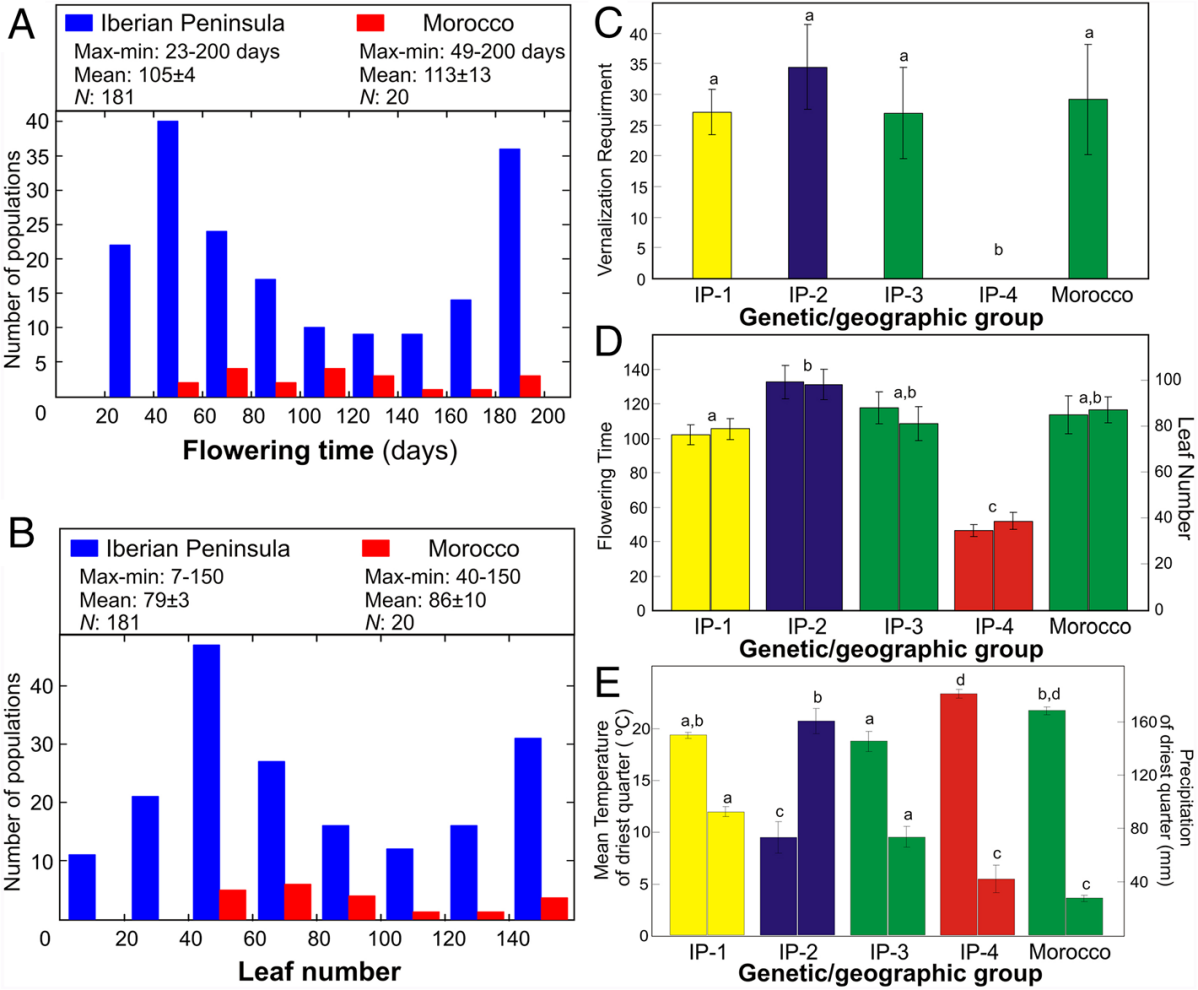
***A. thaliana *flowering and climatic variation in the south-western Mediterranean region. A)** Flowering time and **B)** leaf number of Moroccan and Iberian accessions. **C)** Obligate vernalization requirement, **D)** flowering time (left column) and leaf number (right column), and **E)** mean temperature (left column) and precipitation (right column) of the driest quarter, in the four genetic groups detected with STRUCTURE in the Iberian Peninsula and in Morocco. In **C-E**, bars are the mean ± SE of the accessions assigned to each genetic group, except that group 3 is divided in Iberia (IP-3) and Morocco. Shared or different letters above bars indicate non-significant and significant differences between groups (*P* < 0.05) according to Tukey tests. Bar colors are similar to group colors shown in Figure 2.

The genetic differentiation between Morocco and the Iberian Peninsula was further analysed by measuring three quantitative traits related with flowering induction: vernalization requirement, flowering time and leaf number (Figure 3). Four of the 20 Moroccan accessions (20%) showed an obligate vernalization requirement because they did not flower at all without a low temperature treatment but they flowered after two months at 4°C. This proportion was not significantly different from the 12% frequency observed in Iberia. However, the range of variation among Moroccan accessions for flowering time (49–200 days) and leaf number (40–150 leaves) was significantly smaller than the Iberian variation (23–200 days and 7–150 leaves) (Figure 3A and B). In particular, no Moroccan accession showed an extreme early flowering behaviour, in comparison with 17% of Iberian samples flowering in less than 45 days and with fewer than 32 leaves. Comparisons of flowering traits between Morocco and the four Iberian genetic groups showed that Moroccan accessions were most similar to Iberian group 3, although differences were only significantly different between Morocco and genetic group 4 (Figure 3C and D). In agreement, the genetic differentiation between Morocco and the rest of groups estimated by pair-wise *Q*_*ST*_ values of flowering traits, varied between 0 for groups 1 or 3, and 0.66 for group 4 (*P* < 0.001).

Since *A. thaliana* flowering variation has been involved in climatic adaptation [30] we analyzed if the similarities observed between Morocco and Iberia for flowering traits and genes might be determined by the climatic environment of local populations. Comparisons of climatic variables showed that, on average, Moroccan populations are exposed to higher mean annual temperature (Morocco = 13.5 ± 18.7°C; Iberia = 11.7 ± 26.4°C) and lower annual precipitation than Iberian populations (Morocco = 626 ± 204 mm; Iberia = 652 ± 208 mm; Additional file 2: Table S1). Further comparisons of climatic variables between Morocco and the four Iberian genetic groups showed that the climatic distribution of Moroccan populations was most similar to that of Iberian genetic group 4 distributed in south-western Iberia (Figure 3E). Both, Moroccan populations and Iberian genetic group 4, appeared significantly associated with high temperatures and low summer precipitation. Therefore, the genetic similarity between Morocco and Iberia for flowering genes and traits do not seem to reflect shared adaptations to a similar climatic environment.

### Genetic diversity and structure at a global scale

*A. thaliana* genetic relationships between the south-western Mediterranean region and the rest of world was also studied by analyzing the 201 Moroccan and Iberian accessions together with 136 genotypes representing populations from eleven additional world regions (Additional file 1: Figure S5). Genotyping of these 337 accessions with the 249 segregating SNPs showed that Morocco and the Iberian Peninsula contained the lowest and highest *A. thaliana* diversity, respectively (Table 2).

**Figure 4:**
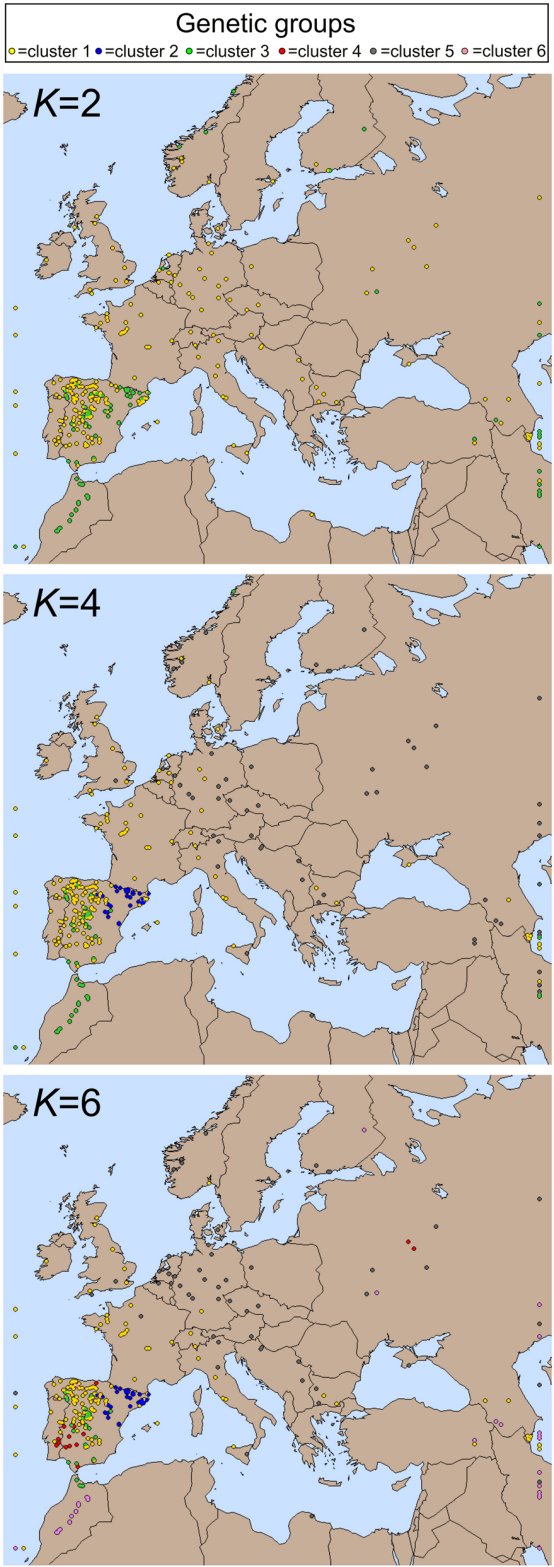
**Genetic and geographic structure of *A. thaliana *at a global scale.** Genetic group assignment of accessions is based on majority membership proportions inferred with STRUCTURE, for *K* = 2, *K* = 4 and *K* = 6. Accessions located outside the Eurasian map are shown at their corresponding latitude in the left (American accessions) and right (Asian accessions) edges.

Analysis of the genetic structure of this *A. thaliana* worldwide collection using STRUCTURE detected six different clusters with distinct geographic patterns (Figure 4, Additional file 1: Figure S6). PCA analysis of the same genotypes found that the first five principal components accounted for 5.0 to 2.0% of the genetic variation. The subsequent clustering of these components detected similar genetic clusters to STRUCTURE (Additional file 1: Figure S7) since 80% of the accessions were assigned to equivalent genetic groups (Additional file 2: Table S2). Four of the genetic groups closely corresponded to the Iberian clusters identified in the previous analysis of the south-western Mediterranean region (Figure 2A, Figure 4). However, two additional clusters (5 and 6) were detected as distributed mainly in eastern Europe, Asia and Africa. Groups 1 and 5 showed the widest geographic distributions in Eurasia. Analysis of the average regional proportions of these groups (Additional file 1: Figure S6) showed that group 1 displays the highest frequency in Iberia and British Isles, and its Eurasian frequency decreases in a west–east direction. By contrast, group 5 displayed high frequency in Central, North and East Europe but it was nearly absent in Iberia and Morocco. Groups 6 and 4 showed an intermediate distribution, with group 6 appearing mainly in Asia and North Africa, while group 4 showed moderate frequency in Asia and East Europe. Groups 2 and 3 displayed restricted geographic distributions since group 3 was present almost exclusively in North Africa and Iberia, and group 2 mostly in the Iberian Peninsula.

*F*_*ST*_ analyses among the six genetic groups rendered an average genetic differentiation of 0.22, but again group 3 displayed the largest differentiations from the rest of clusters (0.24 < *F*_*ST*_ < 0.42; *P* < 0.001) and the lowest genetic diversity (Table 3). In agreement, group 3 was also the first cluster differentiated in the *K* = 2 analysis of STRUCTURE, and an identical cluster was detected in subsequent analyses from *K* = 2 to *K* = 4. This group contained the same 24 Iberian accessions and all 20 Moroccan accessions assigned to group 3 in the previous Mediterranean analysis. However, three and 16 Moroccan accessions were assigned to an Asian group in STRUCTURE analyses with *K* = 5 and *K* = 6, respectively. Thus, global analyses suggest that Moroccan populations of *A. thaliana* are closely related to those from Iberia but also weakly related to Asian populations.

## Discussion

### Low genetic diversity and strong geographic structure in Morocco reflect a homogeneous native history of *A. thaliana* in North Africa

In this study we have systematically sampled and analysed *A. thaliana* populations across Morocco, the southern Mediterranean limit of the species range. Populations were typically found at altitudes between 1000 and 2000 m.a.s.l. in the High Atlas, Middle Atlas and Riff mountains where natural vegetation still predominates (Additional file 1: Figure S1). This region contained less genetic variation than other world regions, for neutral markers, flowering genes and potentially adaptive quantitative traits. In particular, no extreme early flowering accession was found in Morocco, which was in agreement with the association of such flowering behavior with low altitude [30]. The amount of variation detected within Moroccan local populations was lower than that described for southern European Peninsulas proposed as Mediterranean glacial refugia [15,16]. Instead, the average intrapopulation variation of Morocco was similar to that estimated in Scandinavia, the northern limit of the native range reached after postglacial colonization [18,20], or in Japan, a non-native region recently colonized [23].

The strong *A. thaliana* geographic structure found in Morocco at different spatial scales supports an old history as part of the native range. First, populations appeared highly differentiated, the average *F*_*ST*_ value of 0.81 estimated among Moroccan populations being similar to that reported in the northern limit of the species native range [18,20]. Second, in contrast to recently colonized regions such as Japan and North America [23,24] the strong pattern of isolation by distance across Morocco indicates limited geographic seed dispersal from local populations throughout Morocco. However, discrete genetic groups that are geographically structured were also detected consistently by different clustering methods. This indicates the presence of some spatial discontinuities in Morocco IBD pattern, which are likely caused by geographical (physical or environmental) barriers that further limit *A. thaliana* dispersal and contribute to subregional differentiation in Morocco. It must be emphasized that the detection of these clusters is not the result of uneven geographic sampling, as illustrated by the assignment of two populations from the South Middle Atlas subregion, to the genetic cluster of the High Atlas populations (Figure 1). In agreement with the central-marginal hypothesis for the geographic distribution of diversity across species’ ranges [27] Morocco region appears as a trailing edge of *A. thaliana*’s native range characterized by little recent admixture and genetically isolated and differentiated populations. This pattern of variation is likely to be determined not only by contemporary local adaptation of populations to patches of suitable habitat that are close to the species environmental tolerance limits, but also, as discussed below, by abiotic or biotic factors that affected the demographic history of *A. thaliana* in the past [28,29].

### A Moroccan/Iberian specific genetic lineage reveals an *A. thaliana* history shared between south-western Europe and North Africa

The Iberian Peninsula has been proposed as a region containing several *A. thaliana* refugia during the last glaciation, which have played an important role in the postglacial colonization of Europe [9,15,16]. This has been supported by the large genetic diversity of this region and the strong geographic structure reported for multiple kinds of markers and for whole-genome sequences of Iberian accessions [14,16]. In agreement with previous results, our genome-wide SNP analysis finds Iberia as the world region containing the largest diversity, which appears structured into four genetic groups with distinct geographic distributions. Several analyses consistently show that Moroccan populations are genetically related mainly to one particular Iberian group. First, Morocco populations group with Iberian genetic group 3 by clustering analyses of neutral SNP markers. Second, Moroccan populations showed similar flowering behavior to group 3 from the Iberian Peninsula, which is characterized by the absence of early flowering accessions. Third, similar frequencies are found in Morocco and Iberia for the major functional haplotypes described in Europe for flowering genes. Accordingly, Morocco displayed the lowest differentiation from Iberian genetic group 3 for flowering genes and traits, as estimated by *F*_*ST*_ and *Q*_*ST*_ values. Thus, we identified an *A. thaliana* genetic lineage with a shared demographic history in the south-western Mediterranean region.

The Moroccan/Iberian specific distribution of this lineage provides new insights into *A. thaliana* history in North Africa and south-western Europe. On one hand, it indicates that the Strait of Gibraltar has been an *A. thaliana* migration route between the European and African continents. This is in agreement with the well-documented natural history of this region, which recognizes a biodiversity hotspot consisting of two areas with partially overlapping sets of species separated by the Strait of Gibraltar [31-34]. However, our results also suggest that rather limited *A. thaliana* genetic flow has occurred between Morocco and Iberia because this genetic lineage shows an isolated history from the remaining Iberian genetic groups, as indicated by its low genetic diversity and its strong differentiation. In addition, the western Mediterranean distribution of this lineage seems to be predominantly determined by *A. thaliana* demographic history rather than by adaptation to a similar climatic environment in this region. This conclusion is supported by: i) the wide geographic distribution of this group in Iberia and Morocco; ii) the substantial diversity of group 3 for some potentially adaptive haplogroups described for flowering genes such as *CRY2* and *PHYC*; iii) the higher climatic similarity of Moroccan populations with the geographic range of southern Iberia occupied by genetic group 4 than with that occupied by genetic group 3.

On the other hand, the strongly isolated and structured distribution of this genetic lineage in the western Mediterranean region suggests that it was maintained as a refugium during the last glaciations. As reported for several plant and animal species [25], we hypothesize that the Atlas mountain range in north-western Africa could have been a Mediterranean glacial refugium for *A. thaliana* during the Pleistocene. Consequently, we speculate that this genetic lineage might have colonized northern Morocco and southern Spain during the postglacial period. Latitudinal declines in genetic diversity have been associated with recent northward colonization in temperate species [28,35-37]. In agreement with these observations, a weak latitudinal cline of the intrapopulation genetic variation is detected in Morocco, populations from the Riff area containing slightly lower variation than populations from the Atlas Mountains. Furthermore, both neutral markers and flowering genes (*CRY2*, *FLC* and *PHYC*) showed higher private allelic richness in Moroccan than Iberian populations assigned to this genetic lineage. Interestingly, it has been described for several forest plants that colonizations from glacial refugia do not necessarily involve a gradual diversity decrease away from the source populations [29]. In contrast, complex genetic patterns of admixed populations can be generated by contributions from different refugia. Accordingly, the hypothesis of an African Atlas refugium for *A. thaliana* is also compatible with the large genetic diversity and complex structure found in the Iberian Peninsula, since additional *A. thaliana* refugia in this region probably accounted for its overall larger intrapopulation and regional variation. In fact, previous analyses of Iberian intrapopulation variation using the same set of SNPs [38] shows that populations Gra and Mar belonging to genetic group 3 have similar low diversities to Moroccan populations reported here. Nevertheless, we cannot discard that a glacial refugium of this lineage was located in Central Iberia, and that this was the source for the subsequent colonization of north-western Africa.

### Genetic relationships at global scale support a potential migration route between North Africa and Asia

The worldwide genetic and geographic structures detected in this study are in agreement with the previously proposed postglacial colonization routes of Europe during the Pleistocene [9,12,14,17]. In particular, the opposite longitudinal gradients displayed by the frequencies of the two most common genetic groups (Figure 4, clusters 1 and 5) support north-western Iberian and Asian refugia contributing to the colonization of Europe. On the contrary, the restricted geographic distribution of several genetic groups suggests that other Iberian refugia, as well as the newly proposed Moroccan and/or Iberian refugium, did not contribute substantially to such colonization [16]. In addition, the genetic relationship detected between Morocco and Asia, further suggests a potential *A. thaliana* migration route connecting north-western Africa and Asia. Accordingly, a colonization event of Morocco is hypothesized to have occurred from Asia through this northern African route, which could predate the latest glaciations or could have occurred later during the African humid period documented between 15,000 and 5,000 years BP [39].

## Conclusions

The research presented here shows that *A. thaliana* displays low genetic diversity in Morocco but the genetic variation is strongly geographically structured, hence supporting a native history in North Africa. In addition, Morocco appears genetically related mainly to the Iberian Peninsula, which indicates a shared demographic history between south-western Europe and North Africa. Our work illustrates the relevance of systematic population genetic analyses of unknown geographic regions to infer the history of *A. thaliana*. Further comparative studies including underrepresented regions from the rest of North Africa, the Middle East and Central Asia are needed to test the propossed historical hypotheses. In particular, it is likely that the speculated *A. thaliana* refugia trace back to different Pleistocene dates as consequence of strong climatic fluctuations and occurrence of several species retraction/expansion episodes during this period [25]. Eventually, model-based analyses of future whole-genome sequences [14,40] will enable testing of alternative historical scenarios and temporal estimations of demographic events that occurred during *A. thaliana*’s evolutionary history.

## Methods

### Plant material

Twenty *A. thaliana* populations were sampled and geo-referenced during 2005–2009, in the main mountain ranges of Morocco (Figure 1, Additional file 1: Figure S1, Additional file 2: Table S1). Populations were spaced at an average distance of 247 ± 156 km, with a minimum and maximum of 6.5 km and 540 km, respectively. Climatic information from the locations of populations was obtained from http://www.worldclim.org, including monthly, maximum and minimum temperatures, total precipitations and 19 BIO variables [41]. Seeds of one to ten individuals were collected from each population, providing a total of 151 Moroccan samples. Materials are available through the Nottingham Arabidopsis Stock Centre (http://www.arabidopsis.info).

A set of 181 Iberian *A. thaliana* accessions collected from different previously described populations [16,30] and a collection of 136 accessions representing different populations from the rest of the world geographic range were also analysed (Additional file 1:Figure S5, Additional file 2: Table S4).

### Genome-wide SNP genotyping

Accessions were genotyped for a genome-wide set of 343 SNP loci previously selected as frequent polymorphisms in Central Europe (CE; [17]), the Iberian Peninsula (IP; [16,42]), or in worldwide collections (W; [43]) (Additional file 2: Table S5). SNPs were assessed by SNPlex and Veracode methods through the CEGEN genotyping service (http://www.cegen.org). A total of 249 SNPs showing less than 25% missing data (average of 3.9%) were used for population genetic analysis. For the analysis of Morocco genetic diversity, 130 SNPs (17 CE, 35 IP, and 78 W) segregating among the 151 samples were used after removing monomorphic (106 SNPs) or SNPs missing within entire Moroccan population samples (13 SNPs). For analyses of the 201 Moroccan and Iberian populations, 237 polymorphic SNP loci (44 CE, 80 IP, and 113 W) were used after removal of monomorphic loci. For analyses of the 337 Moroccan, Iberian, and worldwide populations, all 249 polymorphic SNPs (55 CE, 80 IP, and 114 W) were included. Genotypic data of all accessions are given in Additional file 2: Table S6.

### Population genetic analyses

Analyses were done with all SNPs and with the three sets of markers separately. Similar results were observed for all sets of markers, indicating that there was no substantial effect on our analyses derived from potential SNP bias ascertainment. Therefore, only results obtained with the total SNP set are shown.

Genetic diversity was measured as number of haplotypes per population (*N*_*H*_), percentage of polymorphic loci (*PL*) and gene diversity (*H*_*S*_) using GenAlex v6.41 [44]. Mean allelic richness (*R*_*S*_) per locus and mean private allelic richness (*Rp*) were estimated with rarefaction to a common sample population size using HP-RARE [45].

Genetic differentiation among populations was estimated by analysis of molecular variance (AMOVA) using Arlequin v3.5.1.2 with multilocus genotypes [46], which calculates *F*_*ST*_-like statistics and their significance from 20,000 permutations.

Isolation by distance (IBD) was tested using Mantel tests for correlation between paired population matrices of geographic distances and genetic distances using Arlequin v3.5.1.2 [47] with 10,000 permutations for significance tests.

Genetic relationships among genotypes were determined by neighbor-joining (NJ) analysis. Genetic distances were measured as pairwise allele differences among Moroccan haplotypes and 10,000 bootstrap permutations were applied to determine the significance of groups using MEGA v5.05 [48].

Discrete clustering of genetic structure was analyzed in three sets of accessions: the 65 different Moroccan haplotypes, the 201 Moroccan and Iberian accessions, and all 337 genotypes of the worldwide collection. Since we were mainly interested in detecting the major genetic groups that were consistently differentiated in each geographic region, we compared two distinct methods. First, genetic structure was inferred using the Bayesian model-based clustering algorithm implemented in STRUCTURE v2.3.3 [49]. Model settings included haploid multilocus genotypes, correlated allele frequencies between populations and a linkage model using genetic distances derived from TAIR10 SNP physical positions (Additional file 2: Table S5). Each run consisted of 50,000 burn-in MCMC iterations and 100,000 MCMC after-burning repetitions for parameter estimation. To determine the *K* number of ancestral populations and the ancestry membership proportions of each individual in each population, the algorithm was run 20 times for each defined number of groups (*K* value) from 1 to 10. The number of distinct genetic clusters was determined by testing the differences between the data likelihood for successive *K* values using Wilcoxon tests for two related samples. The final *K* number was estimated as the largest *K* value with significantly higher likelihood than that of *K*-1 runs (two-sided *P* < 0.005). This was supported by a high similarity among the ancestry membership matrices from different runs of the same *K* value (*H’* > 0.84). The average symmetric similarity coefficient *H’* among runs and the average matrix of ancestry membership proportions, derived from the 20 runs, were calculated with CLUMPP v1 [50].

Population structure was also determined by principal component analysis (PCA) as implemented in the smartpca module of EigenSoft v4.2 [51]. Allele frequency normalization and correction for linkage disequilibrium by regression against both neighbouring SNP markers was used. Significances of explanatory eigenvalues were tested using Tracy-Widom statistics as implemented in the twstats module of EigenSoft. To avoid including axes describing variance unrelated to population structure [51] only the 3 to 5 largest eigenvectors weighted by eigenvalues, each accounting for at least 2% of the variance, were used for subsequent clustering analyses. Distinct clusters were estimated using a hierarchical clustering of Euclidean distances with Ward’s minimized variance as implemented in the R package mclust v3.4.8 [52,53]. Support for the detected clusters was assessed with the relative height values indicative of the genetic distance between clusters. Genetic groups identified by STRUCTURE and PCA were compared by: i) calculating the percentage of accessions assigned to equivalent genetic groups, based on the largest membership proportion estimated for each accession by STRUCTURE and on the cluster assignment determined from PCA; ii) calculating the similarity coefficient *H'* between the STRUCTURE matrix of majority membership proportions and the matrix of cluster assignments derived after PCA.

The average membership proportions of each accession to the different genetic groups and the geographic distribution of the majority population assignments of accessions were plotted using R v2.13 and the R package maps [52].

### Gene sequence analysis

Four flowering time genes known to contribute to the natural variation for flowering traits in *A. thaliana* were sequenced in one individual per Moroccan population: *FRI* and *FLC* involved in the vernalization pathway; *CRY2* and *PhyC* affecting the photoperiod pathway. These genes have been previously studied in world-wide and Iberian collections, where several haplotypes have been associated with flowering and with climatic factors [30,54-56]. The gene regions that were sequenced correspond to the same fragments analysed in a previous Iberian study [30], including the complete *FRI* gene (3.5 kb), 0.7 kb of the first intron of *FLC*, 1.5 kb of *CRY2* coding region and 0.9 kb of *PHYC* promoter. Sequencing was done as previously described [30] using an ABI PRISM 3700 DNA Analyser (Applied Biosystems, Foster City, CA, USA). DNA sequences were aligned using DNASTAR v8.0 (Lasergene, Madison, WI, USA). The 20 Moroccan sequences were analysed together with available sequences from 181 Iberian accessions [30] and nucleotide diversity was estimated using DnaSP v5 [57]. The subset of SNPs in these genes that did not show complete linkage disequilibrium were used to estimate other genetic diversity parameters as described above. Paired genetic distances between groups of accessions were measured as corrected mean number of allele differences and as *F*_*ST*_ values using Arlequin v3.5.1.2. Genbank accession numbers of sequences generated in this study are KF275035-KF275114.

### Flowering initiation analysis

Fifteen plants of one selected accession from each of the 20 Moroccan populations were grown in a glasshouse without vernalization using a randomized experimental design. Flowering initiation was quantified as flowering time (FT) and leaf number (LN) following ref. [30]. FT was measured as the number of days to opening of the first flower. LN was measured as the number of rosette and cauline leaves present at the flowering date. The experiment was continued for 200 days, when the proportion of non-flowering plants per accession was estimated. To avoid underestimation of flowering initiation due to removal of non-flowering individuals, values of 150 leaves and 200 days were given to such individuals, which are the maximum FT value observed in flowering plants and the corresponding LN estimated from the regression line of FT onto LN. Moroccan accessions were also grown and measured after a vernalization treatment of 8 weeks at 4°C to induce flowering [30]. Since all plants flowered in these conditions, the percentage of non-flowering plants without vernalization was taken as a measurement of the obligate vernalization requirement of each accession. Comparison of the flowering behavior between Moroccan and Iberian populations was carried out using available Iberian flowering data obtained in another experiment performed under similar conditions in the same glasshouse [30]. Genetic differentiation between groups of accessions for quantitative traits was measured as *Q*_*ST*_ values estimated from analysis of variance. Between groups (*V*_*B*_) and within groups (*V*_*W*_) variances were estimated by the REML method of variance component analysis, and *Q*_*ST*_ was calculated as *V*_*B*_/(*V*_*B*_ + *V*_*W*_).

## Competing interests

The authors declare that they have no competing interests.

## Authors’ contributions

ACB, JJM-Z, FXP and CA-B conceived the study. AH, FXP, JMM-Z and CA-B collected the samples. BM-V and CA-B generated the laboratory data. ACB, BM-V, FXP and CA-B analysed the data. ACB, FXP and CA-B wrote the paper with the input of all authors. All authors read and approved the final manuscript.

## Supplementary Material

Additional file 1: Figure S1 to Figure S7 **Figure S1.** Moroccan populations surveyed in this study. **Figure S2.** Population structure of *A. thaliana* in Morocco. **Figure S3.** Isolation by distance structure of *A. thaliana* in the south-western Mediterranean region. **Figure S4.** Population structure of *A. thaliana* in the south-western Mediterranean region. **Figure S5.** Geographic distribution of 337 *A. thaliana* populations analysed in this study. **Figure S6.** Population structure of *A. thaliana* at a worldwide scale. **Figure S7.** Genetic and geographic structure of *A. thaliana* at global scale established by PCA. Click here for file

Additional file 2: Table S1 to Table S6 **Table S1.** Geographical and ecological information of Moroccan populations surveyed in this study. **Table S2.** Comparison of STRUCTURE and PCA analyses. **Table S3.** Genetic differentiation between Morocco and Iberian genetic groups for flowering genes. **Table S4.** Worldwide *A. thaliana* accessions analysed in this work. **Table S5.** SNP markers genotyped in this study. **Table S6.** Genotypic data of the 468 accessions for the 249 SNPs analysed in this study. Click here for file
