# Supplementary material for: The genetic structure of Arabidopsis thaliana in the south-western Mediterranean range reveals a shared history between North Africa and southern Europe
Source: BMC Plant Biol. 2014 Jan 10;14:17. doi: 10.1186/1471-2229-14-17 (PMC3890648; doi:10.1186/1471-2229-14-17)

**Additional file 1:** Figure S1 to Figure S7.

**Figure S1.** Moroccan populations surveyed in this study. Each panel shows the landscape of the sampling site, which illustrates the overall ecological features of the populations. **
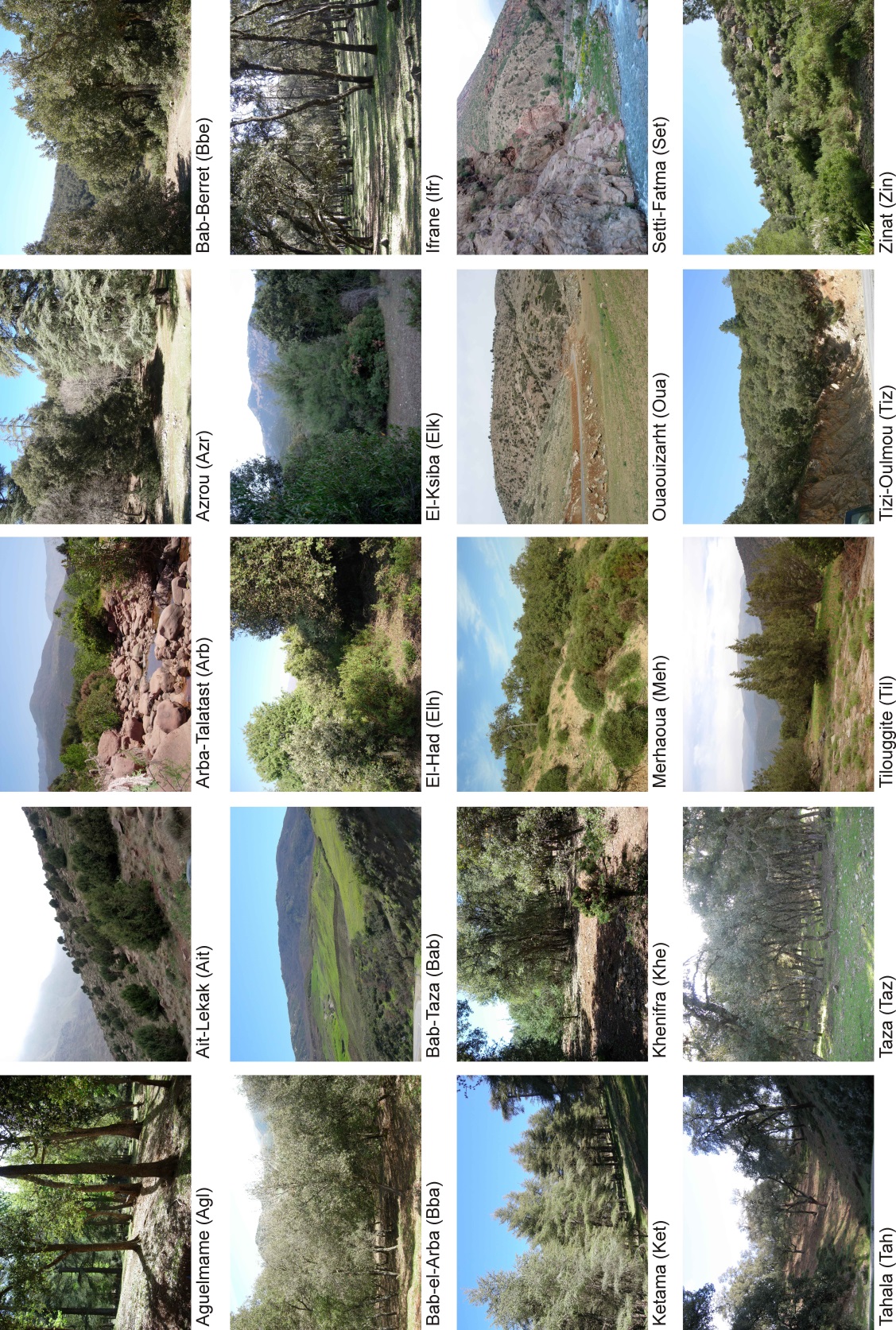
**

**Figure S2.** Population structure of *A. thaliana* in Morocco. A) Unrooted NJ tree constructed from pair-wise proportions of allelic differences among 65 haplotypes from 20 Morocco populations. Percent support >50% from 10,000 bootstraps is shown for each branch node. Branches and haplotypes are coloured according to the majority assignment to the STRUCTURE genetic groups presented in B. B) Genetic relationships among Moroccan haplotypes estimated with STRUCTURE. Each haplotype is depicted as a horizontal bar divided in segments representing the estimated membership proportions of genetic clusters (*K*) fitted in the model. Populations are arranged according to latitude from north to south.

**
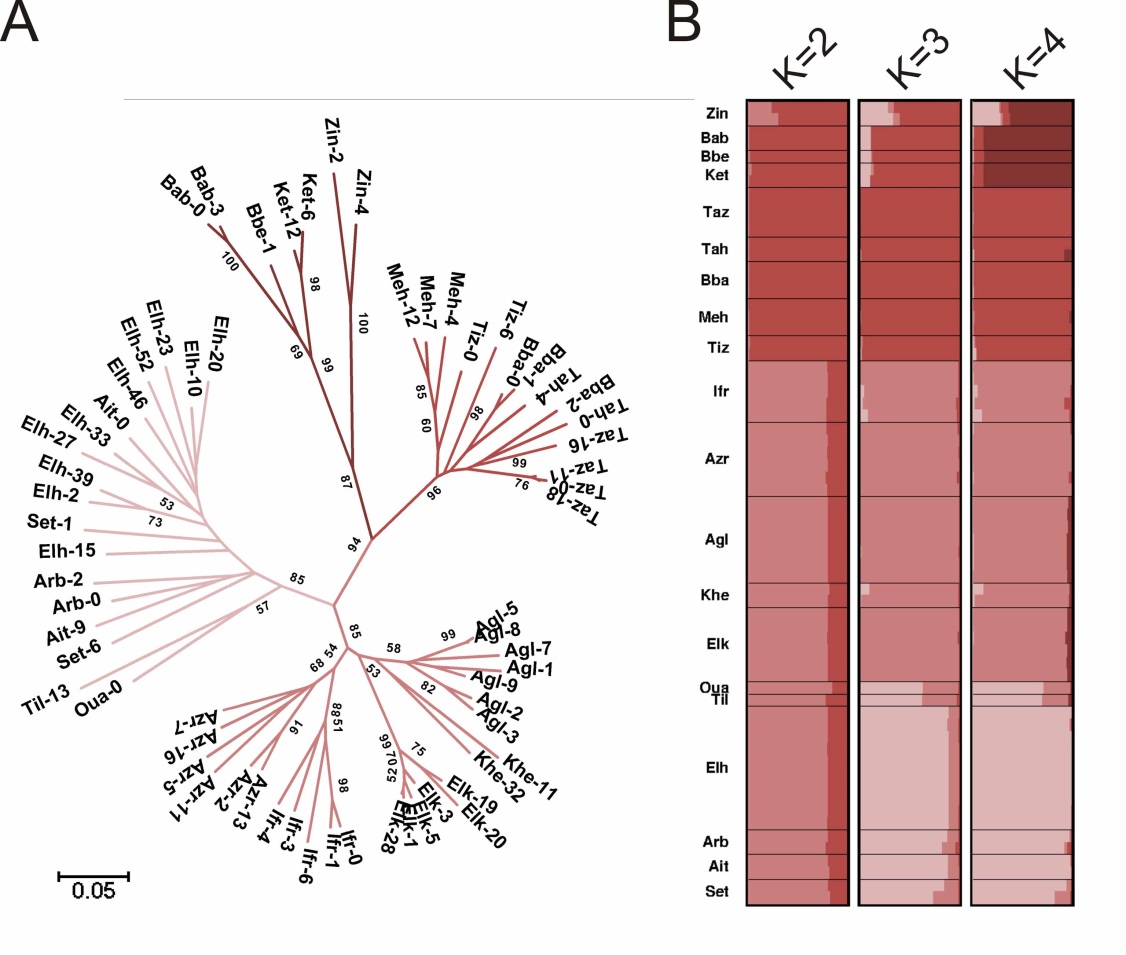
**

**Figure S3.** Isolation by distance structure of *A. thaliana* in the south-western Mediterranean region. Genetic distances are measured as proportion of allele differences between pairs of accessions. Correlation coefficients and slopes of linear regressions for the Iberian Peninsula and Morocco regions are shown above the plot.

**
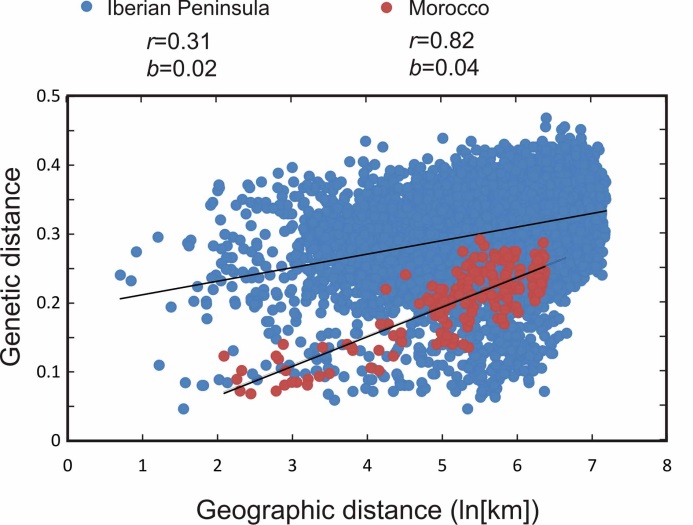
**

**Figure S4.** Population structure of *A. thaliana* in the south-western Mediterranean region. Figure shows the genetic relationships among 20 Moroccan and 181 Iberian individuals from different populations estimated with STRUCTURE. Each individual is depicted as a horizontal bar divided into segments representing the estimated membership proportions of genetic clusters (*K*) fitted in the model. Accessions are arranged and classified (right text line) according to their major membership proportions for four genetic clusters.

**
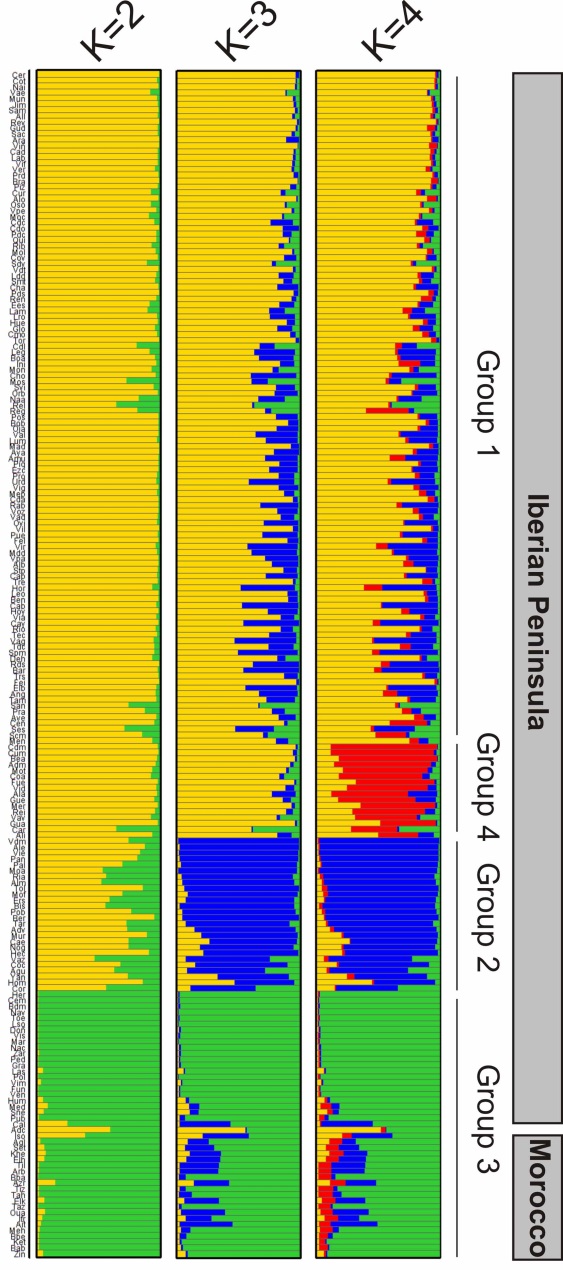
**

**Figure S5.** Geographic distribution of 337 *Arabidopsis thaliana* populations analysed in this study. Left and right panels show locations of North America and Japan, respectively. Central panel shows locations of all other samples coloured according to the following regional groups: red = Morocco; yellow = Iberian Peninsula; green = British Isles; light blue = Central Europe; dark blue = Fennoscandia; purple = South Europe; pink = East Europe; white = West Asia; light grey = Caucasus; dark grey = Central Asia; black = other African samples (Cvi, Can, Mt).


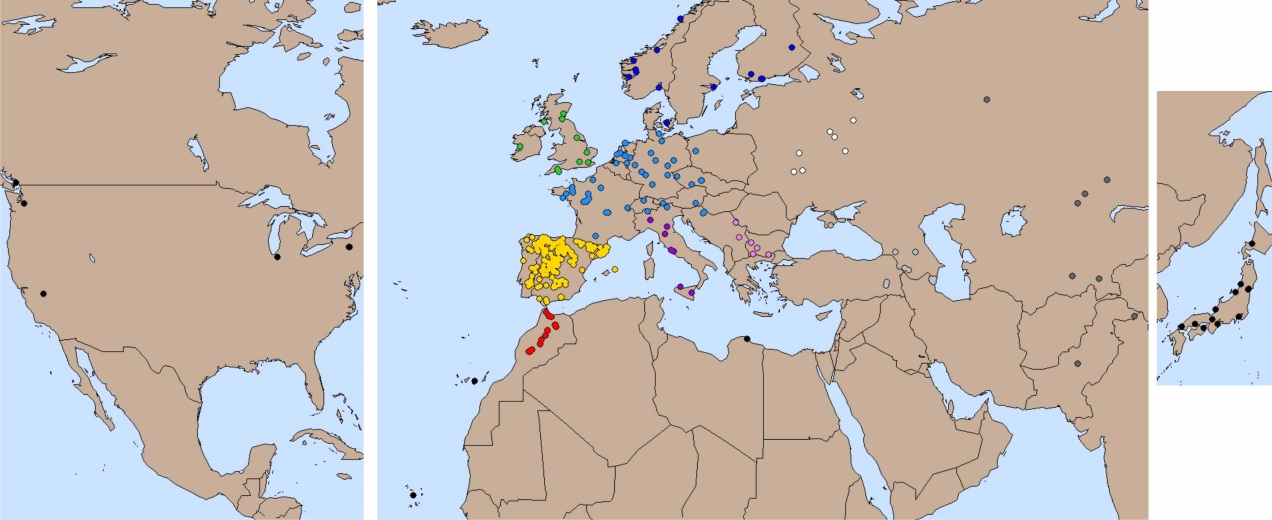


**Figure S6.** Population structure of *A. thaliana* at a global scale. Figure shows the genetic relationships among 20 Moroccan, 181 Iberian and 136 individuals from 11 other world regions estimated with STRUCTURE. Each individual is depicted as a horizontal bar divided into segments representing the estimated membership proportions of genetic clusters (*K*) fitted in the model. Accessions are arranged according to longitude from west to east within geographical regions except for Iberia where they are ordered according to their major membership coefficients for *K*=6. The right panel shows the number of accessions and the mean membership proportions of the six genetic clusters (C1 to C6) in each geographic region.


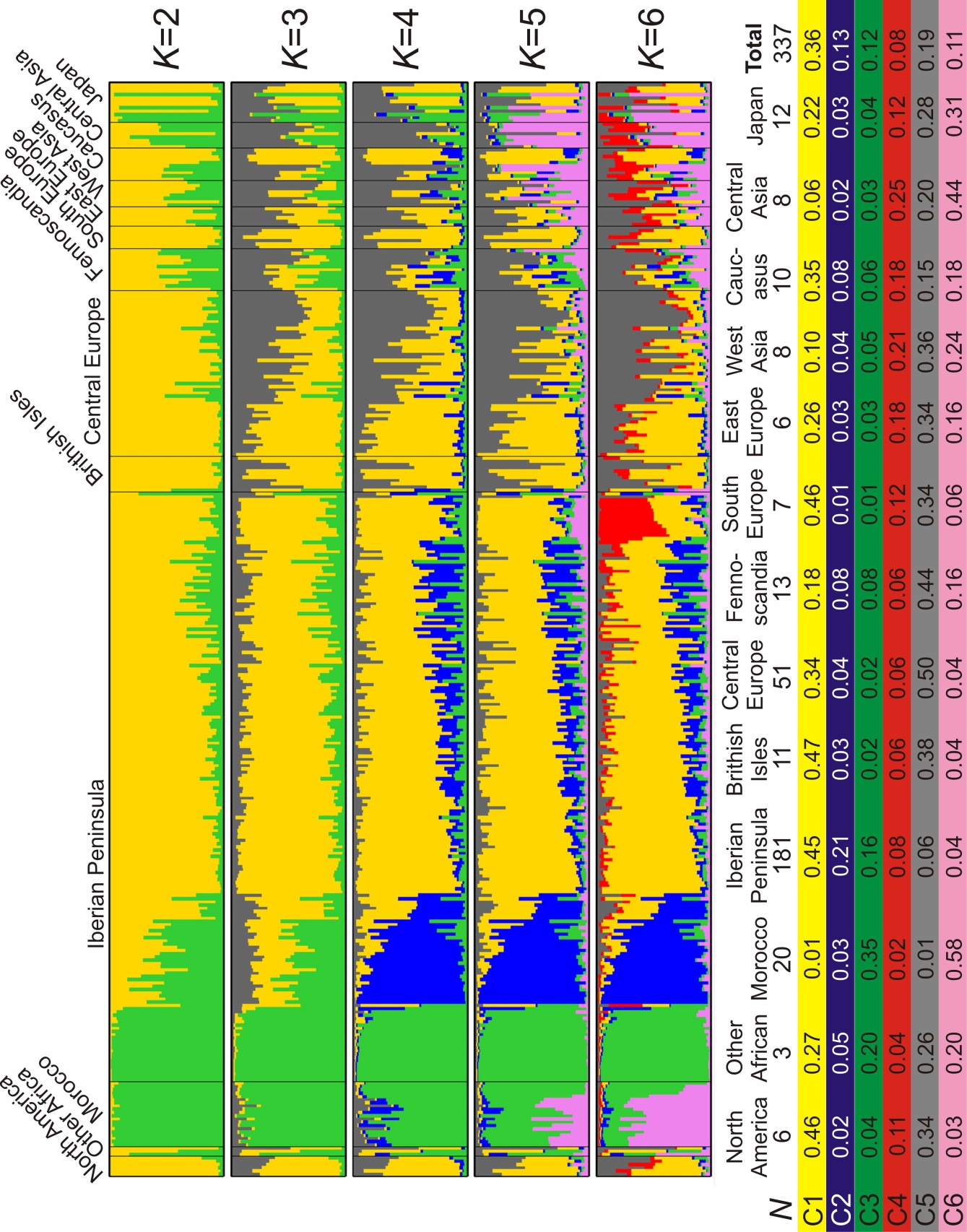


**Figure S7.** Genetic and geographic structure of *A. thaliana* at global scale established by PCA. A) Scatter plots displaying pair combinations of the first three eigenvectors estimated by PCA of the worldwide collection of accessions. The six main groups detected by clustering analysis of the first five principal components are shown with the same colors as the STRUCTURE clusters of Figure 4. B) Geographic distribution of genetic group assignment of accessions based on PCA.


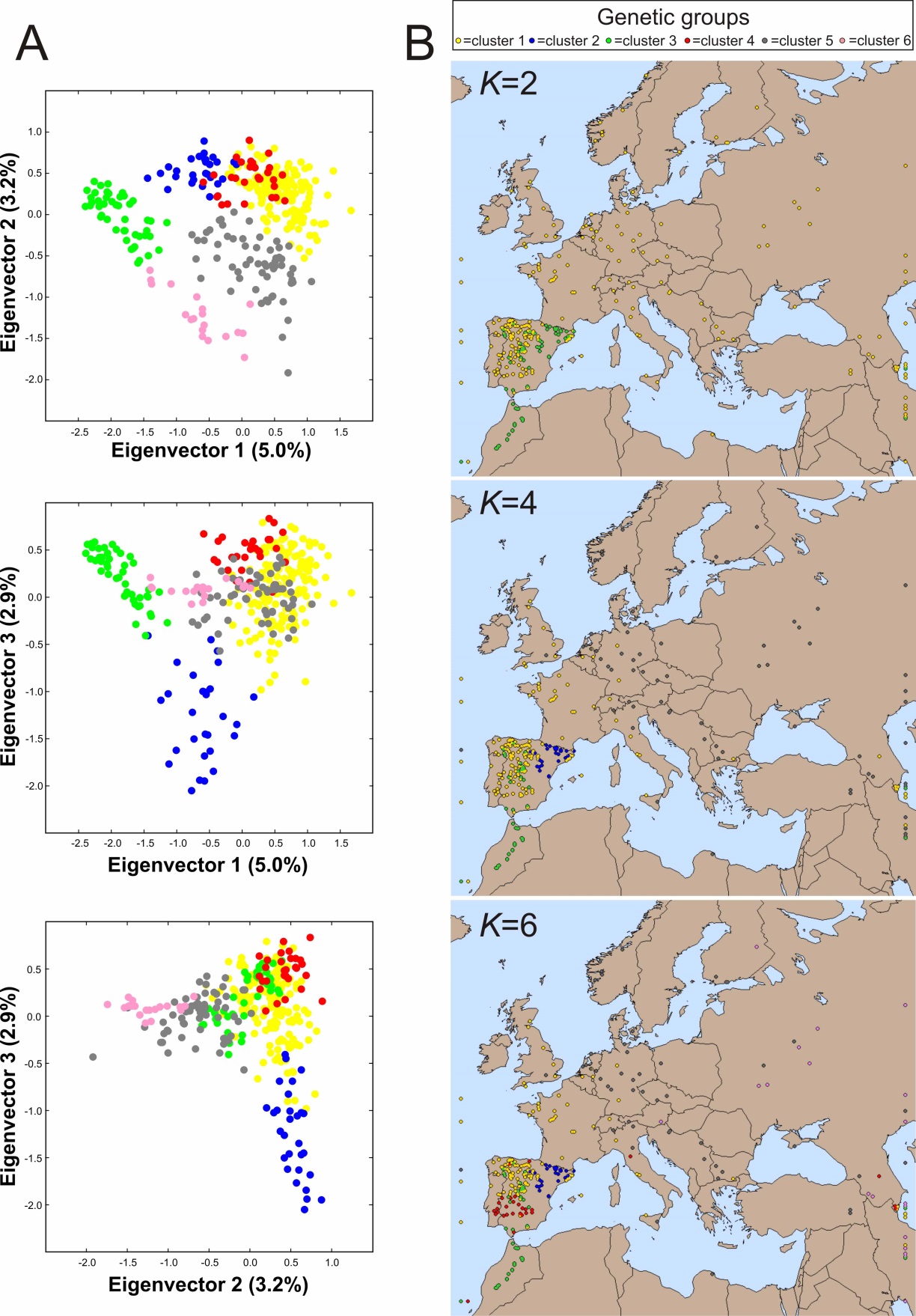

Supplement: Additional file 1: Figure S1 to Figure S7 — Figure S1. Moroccan populations surveyed in this study. Figure S2. Population structure of A. thaliana in Morocco. Figure S3. Isolation by distance structure of A. thaliana in the south-western Mediterranean region. Figure S4. Population structure of A. thaliana in the south-western Mediterranean region. Figure S5. Geographic distribution of 337 A. thaliana populations analysed in this study. Figure S6. Population structure of A. thaliana at a worldwide scale. Figure S7. Genetic and geographic structure of A. thaliana at global scale established by PCA. [file 1471-2229-14-17-S1.docx]
